# Exploring the Use of Virtual Reality for the Delivery and Practice of Stress-Management Exercises

**DOI:** 10.3389/fpsyg.2021.640341

**Published:** 2021-06-03

**Authors:** Desmond Jun Hong Soh, Crystal Huiyi Ong, Qianqian Fan, Denise Ju Ling Seah, Stacey Lee Henderson, Lohsnah Jeevanandam, Kinjal Doshi

**Affiliations:** ^1^Department of Psychology, National University of Singapore, Singapore, Singapore; ^2^Department of Psychology, Singapore General Hospital, Singapore, Singapore

**Keywords:** healthcare professionals, virtual reality, mood, burnout, mindfulness

## Abstract

**Background:**

Mindfulness-based interventions may benefit healthcare professionals with burnout symptoms. Virtual reality (VR) may reduce initial difficulty of engaging in mindfulness exercises and increase participants’ engagement through immersion and presence.

**Aim:**

The aim was to investigate how VR affects participants’ experience of engagement with mindfulness practice, and its impact on quality of practice and negative mood states.

**Methods:**

Fifty-one healthcare professionals were randomized to receive either a visualization or non-visualization mindfulness practice, to compare the quality of practice through the use of audio only vs. with a virtual reality interface. Selected self-reported measures were collected during the session (immersion, quality and difficulty of practice, mood states and likelihood for future practice).

**Results:**

Results showed that order instead of type of modality administered made a difference in quality of mindfulness practice. A greater sense of presence was reported with VR if administered after audio (*F* = 4.810, *p* = 0.033, Partial η^2^ = 0.093). Further, participants described difficulty practicing with audio if administered after VR (*F* = 4.136, *p* = 0.048, Partial η^2^ = 0.081). Additionally, lower mood disturbance was reported with VR if administered after audio (*F* = 8.116, *p* = 0.006, Partial η^2^ = 0.147). Qualitative responses echoed a preference for VR to engage better, in addition to improved mood states after practice.

**Conclusion:**

Findings suggest that VR has the potential to provide healthcare professionals with an alternative or a supplement to conventional mindfulness practice.

## Introduction

Work-related stress is prevalent among healthcare professionals, especially among those who report heavy workloads, long working hours and having to deal with uncooperative patients (Boran et al., 2012). Consequently, healthcare professionals are at risk of emotional exhaustion (Sturgess and Poulsen, 1983) and burnout (Khamisa et al., 2013; Deschamps et al., 2018). High levels of burnout among healthcare professionals could potentially lead to detrimental consequences for both the professionals and their patients. It has been shown to have an adverse effect on their work performance (Barsade, 2002) resulting in medical errors and negligence, which impact the quality of care provided to patients (Panagioti et al., 2018). Thus, it is important for healthcare settings to consider strategies that will address, even prevent, burnout among healthcare professionals.

Mindfulness practice has been increasingly incorporated into clinical settings to prevent burnout and build resilience among healthcare workers (Joyce et al., 2018). Mindfulness looks to increase an individual’s awareness of the present-moment and the acceptance of internal (e.g., thoughts and emotions) and external experiences in a non-judgmental manner (Brown and Ryan, 2003). Healthcare professionals, who experience time constraints, may benefit from short and effective interventions. Brief mindfulness practices have also been found to effectively improve the well-being of healthcare providers (Gilmartin et al., 2017). Healthcare professionals, however, find it difficult to commit to regular mindfulness practice due to their inability to find conducive environments, making it difficult for them to be present with the practice and reap its benefits (Nararro-Haro et al., 2016).

Slater (2009) suggested that when two independent constructs, place illusion and plausibility illusion, occur, users will respond realistically to the VR environment. By combining visual and auditory components, VR creates a sensory illusion that is able to produce a simulation of reality (Biocca and Levy, 1995), thereby creating a sense of presence—the feeling of “being there” in the virtual world (Cooper et al., 2018). This state of immersion is associated with focused attention and an altered sense of time and self (Brown and Cairns, 2004)—qualities which are also aligned with the intended state of individuals during a mindfulness practice (Berkovich-Ohana et al., 2013; Dickenson et al., 2013). The complementary features of virtual reality technology and mindfulness, coupled with the novelty of the medium, may contribute to increased levels of motivation and intention to continue practice, thereby improving overall engagement in mindfulness-based interventions among healthcare professionals.

The limited research investigating the combination of VR with mindfulness training found higher reported levels of mindfulness and lower levels of negative emotions when incorporating VR into mindfulness practice (Navarro-Haro et al., 2017). However, these studies were conducted with mindfulness experts, who experience different effects of mindfulness compared to novice meditators (Keune and Perczel Forintos, 2010; Taylor et al., 2011). This pilot study proposes to investigate quality of mindfulness practice with novice meditators using VR and hypothesizes that VR mindfulness will offer higher levels of engagement and presence. Furthermore, this study examines the impact of the brief mindfulness intervention delivered using VR on their subjective experiences of negative emotions.

## Materials and Methods

### Participants

Healthcare professionals from several medical institutions were recruited through letters of invitation sent via e-mail. Those who had prior experience with mindfulness, meditation or had any medical condition that will be triggered or worsened by using the virtual reality headset were excluded from the study. Participants included in the study were above 21 years of age and fluent in English. They were compensated with a $10 voucher upon completion of the session. Ethics approval was obtained from the SingHealth Centralised Institutional Review Board (CIRB: 2019/2381).

### Study Design and Procedure

**FIGURE 1:**
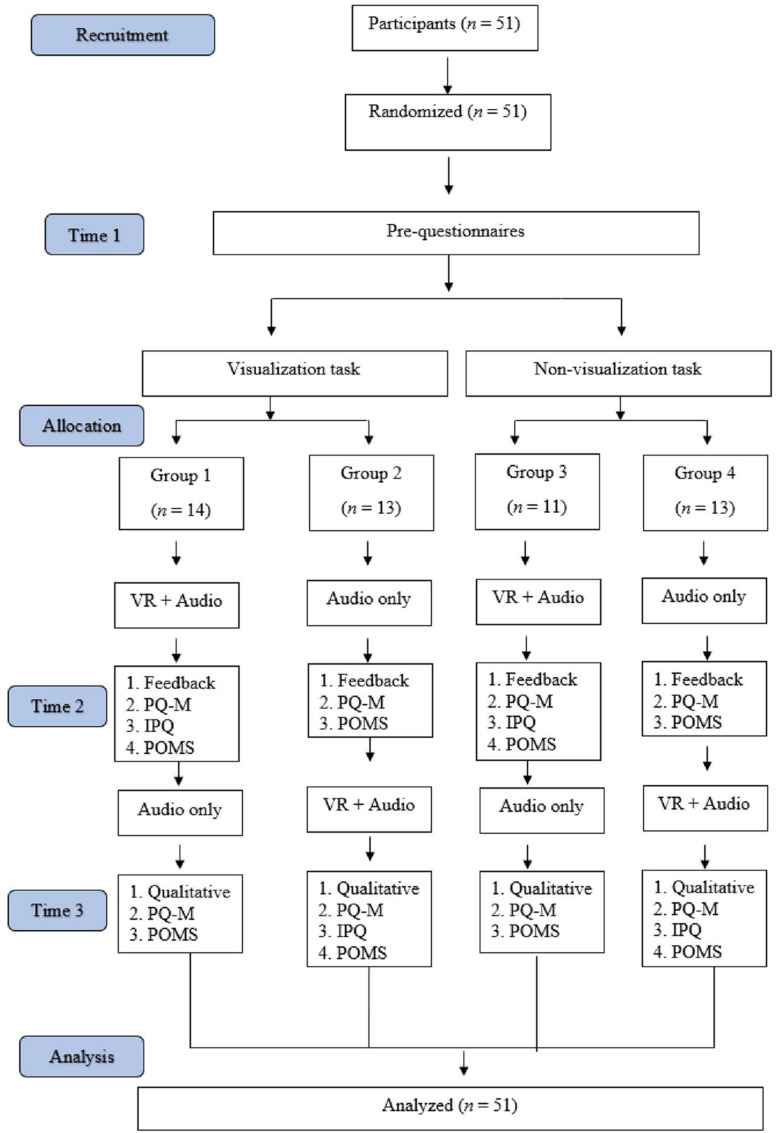
Study consort diagram.

This study employed a randomized controlled design (Figure 1). Prior to the start of the study, participants were required to read through a Participant Information Sheet which warned about the potential risk for cybersickness discomfort and informed them that they could withdraw from the study at any point in time. Participants who signed the Informed Consent Form were randomly assigned to one of four groups using a randomization protocol; participants either experienced the guided mindfulness practice with the use of VR first, of two exposures during the session, or vice versa—where they experienced it without the use of VR first of two times. This was done to reduce an order bias influencing their preference as we investigated their preference between audio only or VR. Therefore, we counter balanced which versions participants received. A random number generator was used to generate numbers between one and four, with participants being assigned to the corresponding group on recruitment (Randomness and Integrity Services Ltd., n.d. *Random.org*)^1^. Participants were required to attend one session, which lasted approximately an hour, and data was collected over three time points during the session: at baseline, after the first exposure to guided mindfulness and following the second exposure to guided mindfulness. Each exposure of guided mindfulness with and without the virtual reality set-up was 10 min in duration (Figure 1).

### Virtual Reality Intervention Set-Up

**FIGURE 2:**
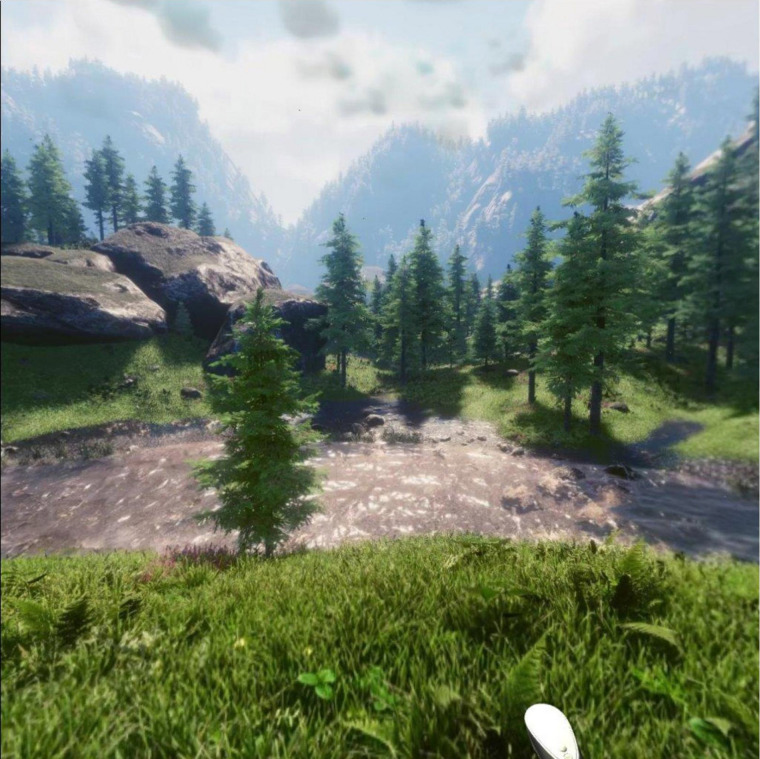
Screenshot of VR environment used.

Participants were assisted with putting on the VR headset and given earphones for listening to the audio guide of the mindfulness practice. An Oculus Go headset was used to simulate the virtual reality environment. The headset ran a program called Guided Meditation VR^TM^ (Cubicle Ninjas LLC, 2016). Approval was sought from the developers for the use of their program in this study. This program placed the participant into a simulated nature environment that consisted of trees, grass, mountains and a stream (Figure 2 for screenshot); the guided audio tracks available with this program were not used as the investigators did not classify them as guided mindfulness practices, and further, to control the length and nature of the practices.

The headset was activated only when participants were engaged in the “VR and Audio” intervention and was de-activated when they were engaged in the “Audio Only” intervention. For the “Audio Only” intervention, participants were advised to close their eyes while wearing the headset, and if they found it uncomfortable, to have them open with nothing to view. Use of the headset during the “Audio Only” intervention was intentional to account for any physical effect difference.

Participants were tasked to listen to a guided mindfulness practice audio for 10 min delivered in stereo to both ears through earphones. The visualization practice invited participants to visualize placing their thoughts on individual leaves floating down a stream (Leaves On Stream—LOS) (i.e., Leaves on the stream) (Didonna, 2009). The non-visualization practice invited participants to notice their thoughts as they come and to let them go (Thought Noticing Exercise—TNE) (McCown et al., 2010).

### Materials

Demographic questionnaires consisted of questions about the participants’ age, ethnicity, gender, and profession.

The Depression Anxiety and Stress Scales-21 (DASS-21; Lovibond and Lovibond, 1995) is a 21-item questionnaire that measures three related negative emotional states of depression, anxiety and stress. A higher total score for each domain indicates higher severity levels. Internal consistency alpha coefficients range from 0.76 to 0.91 (Lovibond and Lovibond, 1995).

The revised six-item Practice Quality-Mindfulness Questionnaire (PQ-M; Re et al., 2013) is a 6-item questionnaire that measures the quality of mindfulness practice. It measures three different factors: (a) Overall quality of practice, (b) Present-moment attention, and (c) Receptivity (i.e., willingness to experience what is arising in the formal practice). Internal consistency alpha coefficients range from 0.66 to 0.87 (Re et al., 2013).

The igroup Presence Questionnaire (only after VR) (IPQ; Schubert et al., 2001) is a 14-item questionnaire that measures the level of presence an individual experiences within a virtual reality environment. It measures four different factors: (a) Spatial Presence, (b) Involvement, (c) Experienced realism and (d) General presence “g.” However, for the purpose of this study and relevance to quality of practice, only the General Presence (the “sense of being there”) subscale was utilized. It is important to note that this subscale item has strong factor loadings on all three other subscales (Schubert et al., 2001). Internal consistency alpha coefficients range from 0.81 to 0.85 for the different factors in the questionnaire, e.g., Spatial Presence, Involvement and Experience Realism (Schwind et al., 2019).

The abbreviated Profile of Mood States (POMS; Grove and Prapavessis, 1992) is adapted from the original 65-item POMS scale and consists of 40 items assessing seven different subscales of transient mood states. The subscales of positive mood states include (a) vigor and (b) esteem-related mood, and the subscales of negative mood states include (a) tension, (b) anger, (c) fatigue, (d) confusion, and (e) depression. Each question is scored from 0 (not at all) to 4 (extremely). The Total Mood Disturbance (TMD) score is computed by subtracting the scores of the two positive dimensions from the subtotal score of the five negative dimensions, and then adding 100. Higher scores indicate greater mood disturbance. The abbreviated POMS has moderate to high internal consistency, with Cronbach’s scores for the subscales ranging from 0.66 to 0.95 (Grove and Prapavessis, 1992).

Participants were also tasked to complete a feedback questionnaire designed by the investigators to understand their experiences with the mindfulness practices. The questionnaire covered queries about the difficulty of the practice and the likelihood of practicing mindfulness in the future. Participants were asked to provide qualitative responses to two open-ended feedback questions. First, to describe their experience after each practice with the different modes of delivery (i.e., VR and Audio). Second, to describe any challenges encountered during each practice.

### Data Analysis

The statistical analysis was performed using statistical software IBM SPSS Statistics 25. A manipulation check using Analysis of Variance (ANOVA) and Chi-Square Test was performed to check randomization. Repeated measures ANOVA was conducted to investigate effects of modality (VR vs. Audio) and practices (LOS vs. TNE) on outcome measures (i.e., quality of mindfulness practice, difficulty of practice and likelihood of engaging in future practice, and mood disturbance). Two-way between-subjects ANOVA was conducted to examine effects of type of practice (LOS vs. TNE) and order of practice (VR first vs. Audio first) on outcome measures above and experience within a virtual reality environment.

A preliminary analysis of reading through a sample of qualitative responses was performed. The decision to include responses from the open-ended questions was made as it: (a) contributed valuable insight to the quantitative responses and (b) included many individual challenges and expression of emotions when introduced to this novel practice. Responses were analyzed as a combination framework approach of inductive open coding and content analysis. Codes were generated independently by one of the authors (SH) and arbitrated by a second author (KD) for rigor. Analysis of the quotes in the feedback questionnaire involved quantifying the frequency of keywords that participants used in their responses.

## Results

### Demographic Variables

Fifty-one healthcare professionals participated in this study [Age*M* = 29.82, *SD* = 6.70; male = 10 (19.6%)]. They included medical doctors and students, nurses, and various allied health professionals (i.e., educational, occupational and speech therapists, medical social workers, and psychologists). Out of 51 participants, 88.2% were Chinese, 5.9% were Indian, 2.0% were Malay, and the remaining 3.9% identified as “others.”

### Manipulation Check

A manipulation check using ANOVA for continuous variables and chi-square test for categorical variables was conducted to assess for significant group differences prior to the start of the session. Results of ANOVA showed that there were no significant group differences (LOS VR first vs. LOS audio first vs. TNE VR first vs. TNE audio first) in age (*F* = 0.103, *p* = 0.958), three DASS subscale scores (depression: *F* = 1.255, *p* = 0.301; anxiety: *F* = 0.259, *p* = 0.854; and stress: *F* = 0.826, *p* = 0.486), and mood disturbance before practice (*F* = 1.667, *p* = 0.187). Results of Chi-Square Test showed no significant group difference in gender (chi sq = 0.290, *p* = 0.962), ethnicity (chi sq = 9.276, *p* = 0.412), and profession (chi sq = 19.029, *p* = 0.583).

### Results of Repeated-Measures ANOVA on Quality of Practice

Two-way repeated-measures ANOVA analysis (Modality of Practice ^∗^ Guided Practice) was conducted to examine effects of modality: VR vs. Audio, and guided practices: LOS vs. TNE on quality of practice (Table 1). Assumptions of repeated-measures ANOVA analysis were tested. Normality assumption holds as indicated by skewness and kurtosis, in combination with Q-Q plot. It was found that all the skewness and kurtosis values of these repeated-measures ANOVA tests were within the range from -1 to 1, except for kurtosis of difficulty for practice with audio was -1.147. According to the Q-Q plot, all the data points were well-distributed along the diagonal line, indicating a normal distribution. In addition, sphericity assumption was met as repeated-measures variables have only two levels, VR vs. audio. Therefore, no correction was made in the analysis. There was no main effect of practice modality on receptivity (*F* = 3.827, *p* = 0.056, Partial η^2^ = 0.072). However, this result implied a tendency that participants may be more willing to experience what arose during the practice with VR (*M* = 78.23, *SD* = 14.38) vs. audio (*M* = 73.15, *SD* = 19.81). Additionally, there was no significant effect of practice modality on attention, difficulty in practice, and likelihood of engaging in future practice (Table 1). Since there were no significant interaction effects found in these analyses, no *post-hoc* analyses were conducted.

**TABLE 1:** Results of repeated-measures ANOVA analysis.

		*F*	*p*	Partial η^2^
Attention	Main effect of modality	0.031	0.861	0.001
	Main effect of practice	0.408	0.526	0.008
	Interaction effect	0.004	0.947	0.000
Receptivity	Main effect of modality	3.827	0.056	0.072
	Main effect of practice	1.422	0.239	0.028
	Interaction effect	0.963	0.331	0.019
Difficulty	Main effect of modality	0.337	0.564	0.007
	Main effect of practice	0.527	0.471	0.011
	Interaction effect	1.332	0.254	0.026
Future likelihood	Main effect of modality	2.672	0.109	0.052
	Main effect of practice	1.518	0.224	0.030
	Interaction effect	0.787	0.379	0.016
Mood disturbance	Main effect of modality	2.653	0.110	0.051
	Main effect of practice	3.520	0.067	0.067
	Interaction effect	0.241	0.626	0.005

### Results of Two-Way Between-Subjects ANOVA on Quality of Practice

Next, order of practice was analyzed. Two-way Between-Subjects ANOVA was conducted to examine effects of type of practice (LOS vs. TNE) and order of practice (VR first vs. Audio first) on quality of practice (Table 2). Results of normality assumptions were reported in 3.3. Homogeneity assumption of error variance were tested by Levene’s Test and the results were included in Table 2. Results of two-way ANOVA showed a significant main effect of order on presence (*F* = 4.810, *p* = 0.033, Partial η^2^ = 0.093) and main effect of type of practice on realism for practice with VR (*F* = 5.661, *p* = 0.021, Partial η^2^ = 0.108). Specifically, participants reported a higher sense of being physically present in the virtual environment while engaged in the mindfulness practice if they did it using VR after practicing it with only audio (presence Audio first: *M* = 4.08, *SD* = 1.10), compared to having done it without the prior experience of audio (presence VR first: *M* = 3.45, *SD* = 1.01). Participants also experienced preference for realism with LOS practice (*M* = 3.06, *SD* = 0.77) compared to TNE (*M* = 2.58, *SD* = 0.61) practice with VR. For practice with audio, there was main effect of order in difficulty doing the practice with audio only (*F* = 4.136, *p* = 0.048, Partial η^2^ = 0.081), indicating that participants reported more difficulty doing audio practice if it was done after VR practice (*M* = 5.08, *SD* = 2.43) compared to audio before VR (*M* = 3.65, *SD* = 2.37). There were no significant interaction effects found in these analyses, so no *post-hoc* analyses were conducted.

**TABLE 2:** Results of two-way between-subjects ANOVA analysis.

	Homogeneity test (*p*)		*F*	*p*	Partial η^2^
VR_Attention	0.330	Main effect of practice type	0.312	0.579	0.007
		Main effect of order	0.000	0.987	0.000
		Interaction effect	0.173	0.679	0.004
VR_Receptivity	0.675	Main effect of practice type	0.402	0.529	0.008
		Main effect of order	0.428	0.516	0.009
		Interaction effect	0.005	0.944	0.000
VR_Difficulty	0.199	Main effect of practice type	2.024	0.161	0.041
		Main effect of order	0.500	0.483	0.011
		Interaction effect	1.653	0.205	0.034
VR_Likelihood	0.460	Main effect of practice type	0.232	0.632	0.005
		Main effect of order	0.223	0.639	0.005
		Interaction effect	0.000	0.990	0.000
VR_Presence	0.698	Main effect of practice type	1.996	0.164	0.041
		Main effect of order	4.810	0.033*	0.093
		Interaction effect	0.020	0.889	0.000
VR_Involvement	0.939	Main effect of practice type	0.990	0.325	0.021
		Main effect of order	1.014	0.319	0.021
		Interaction effect	0.780	0.382	0.016
VR_Realism	0.697	Main effect of practice type	5.661	0.021*	0.108
		Main effect of order	0.010	0.919	0.000
		Interaction effect	1.537	0.221	0.032
VR_Immersion	0.617	Main effect of practice type	0.785	0.380	0.016
		Main effect of order	0.144	0.706	0.003
		Interaction effect	0.680	0.414	0.014
VR_Mood	0.492	Main effect of practice type	3.316	0.075	0.066
		Main effect of order	8.116	0.006**	0.147
		Interaction effect	0.001	0.973	0.000
AUD_Attention	0.771	Main effect of practice type	0.317	0.576	0.007
		Main effect of order	1.014	0.291	0.024
		Interaction effect	0.244	0.624	0.005
AUD_Receptivity	0.283	Main effect of practice type	1.771	0.190	0.036
		Main effect of order	0.166	0.686	0.004
		Interaction effect	0.347	0.559	0.007
AUD_Difficulty	0.359	Main effect of practice type	0.002	0.968	0.000
		Main effect of order	4.136	0.048*	0.081
		Interaction effect	1.460	0.233	0.030
AUD_Likelihood	0.435	Main effect of practice type	2.440	0.125	0.049
		Main effect of order	0.213	0.647	0.005
		Interaction effect	0.023	0.881	0.000
AUD_Mood	0.580	Main effect of practice type	4.047	0.050*	0.079
		Main effect of order	1.685	0.201	0.035
		Interaction effect	0.021	0.885	0.000

*p< 0.05, **p < 0.01.

### Results in Mood Disturbance

There was no effect of practice type on mood disturbance (*F* = 3.520, *p* = 0.067, Partial η^2^ = 0.067) (Table 1). There was a significant main effect of order on mood disturbance (*F* = 8.116, *p* = 0.006, Partial η^2^ = 0.147) for VR practice. This indicated that participants reported lower mood disturbance in VR practice if VR was administered after audio (Audio first: *M* = 84.85, *SD* = 8.68) compared to before audio (VR first: *M* = 91.96, *SD* = 9.31).

### Feedback Questionnaire

Qualitative data collected from the feedback questionnaire revealed that participants were able to engage more easily with the practice using a VR headset. However, participants also expressed suggestions for improvement due to discomfort and shared their fluctuations in mood as a result of participating in the practices with VR.

#### Engagement

Three participants iterated the ability to draw awareness to their breath more easily with just the audio guide, “*The audio helped me to be more mindful of my breathing*” (P15). However, when introduced to the VR modality, 23 participants noted that there was a sense of visual immersion and engagement with VR. They were better able to engage visually and indicated a preference for immersion into the practice using VR, “*Having visual imagery helped in being present as compared to having sounds alone.*” (P29), “*The nice scenery helped me to distract myself away from actual world/surroundings and focus on audio instructions*” (P38). Engagement in the visualization exercise was easier with VR when compared to audio, “*Having the presence of VR + the soundtrack made it easier for me to imagine the river/leaves hence it allowed me to participate more/engage more in the activity. I could even follow through as my ‘leaves’ flowed downstream*” (P21).

The immersive experience created a sense of *Presence* with the practice. Four participants were able to sustain focus, “*I preferred the VR experience. The visual helped me to stay present but was not too distracting*” (P39), “*without visual guidance/VR*—*easily distracted by my own thoughts (more occurrences) as there is no visual image to help focus on*” (P23). However, one participant found the visual experience restrictive, “*Easier at the start compared to the VR. Could imagine leaves being larger and fitting into my stream image as opposed to the predefined VR one*” (P2).

#### Suggestions for Improvement to VR Experience

While using a VR headset improved engagement with the practice, 12 participants expressed physical discomfort with the equipment. It was either too heavy or they found the environment not as realistic, “*I prefer without the VR as the headset after a while felt a bit restrictive with my sense of space. Also the imagery was not very sharp so it was not enough to sustain my alertness for a long period of time. The colors of the VR are unable to replicate the naturalness outside*,” (P28). The quality of the virtual atmosphere as well as comfort is pertinent to the immersive experience. Participants suggested to ensure making the room comfortable as well, “*want to have a reclined chair to fully relax as the device is heavy on my head*” (P28). A single participant on the other hand had discomfort issues with the earpieces, “*the ear pieces kept sliding out of my ears*” (P11).

Participants found it “*hard to stay focused between silences*” (P51). The pauses and silences allowed for individual practice between the audio guided instructions created a disconnection and loss of focus with the practice. 11 participants had similar suggestions to replace the silences with nature sounds or subtle music to aid with their distraction, “*The voice recording was very soothing, but the breaks could have been replaced by white noise”* (P3).

#### Mood

Although there were several challenges with the use of the VR, most participants were new to the experience. Experiencing mindfulness for the first time, 13 participants reported feeling fatigued after the practice, “*Staying awake for the 10* min *while on the audio was difficult*” (P36). Notably, they were already exhausted and insomnolent coming into the session after their work shift, “*I really wanted to just close my eyes and sleep. I am very sleep deprived*” (P47). Post VR session, 18 participants reported feeling relaxed and calm after completing the practice, “*Nevertheless, it was still a relaxing calming experience allowing my thoughts to surface and subside organically*” (P44).

## Discussion

This pilot study highlighted virtual reality as a modality to provide an immersive environment that promotes engagement in mindfulness practice among novice practitioners. While there were no significant effects of VR on the quality of mindfulness practice in one session, participants reported that using just audio for practicing mindfulness was more difficult after having experienced practicing in VR. Furthermore, individuals also acknowledged a reduction in their negative mood states after engaging in a brief mindfulness practice using virtual reality if administered after audio only.

The ability to immerse into the practice using virtual reality may have allowed participants to feel more present while doing the practice. Attention Restoration Theory (ART) proposes that the act of intentionally directing attention induces fatigue that affects several issues including mental health and that the recovery of attentional resources is facilitated by interactions with the environment (Kaplan, 2001). For an environment to be considered restorative, it requires properties that include: (a) feeling like the individual is “away” from his/her daily routine environment; (b) having environmental features that grab your attention with little effort through fascination; (c) having a variety of environmental features that keep the individual immersed; and (d) compatibility with the individual’s preference of environment (Kaplan, 2001; Seabrook et al., 2020). Our results highlighting the order effect of a lower mood disturbance through practicing mindfulness in VR if administered after audio only is interesting and warrants further investigation in future studies. As participants also reported being more “physically present” in VR after audio only practice, one possible explanation is that the aforementioned restorative properties of the VR environment facilitated the participant’s mindfulness practice, resulting in a lower mood disturbance.

Guided imagery practices in the context of a nature environment have been suggested to be more effective as it provides a richer multi-sensory scenery to engage in Brymer et al. (2014) and Nguyen and Brymer (2018). Further, participants were more immersed when engaged in the leaves on the stream exercise as compared to the thoughts noticing exercise, which highlights the importance of visual relevance of the practice for the novice practitioners. This may be an important consideration for novice practitioners as our results suggests that the level of immersion was enhanced through the visualization practice. The leaves on the stream practice required the individual to conjure up images that were congruent with the virtual environment, and therefore, lowered the effort needed to visualize for the activity. On a separate note, engaging in a mindfulness practice within a visual environment of nature alone may be therapeutic (Schutte and Malouff, 2018). Future studies need to evaluate the qualities and types of virtual environments that enable immersion, to create a conducive environment for mindfulness practice.

Interestingly, the audio component was also important in creating a sense of realism, which helped participants to be engaged in the practice. Participants noted that the pauses and silences in the audio track disconnected them from the practice and caused them to lose focus, especially given that the visual environment was not accompanied by a congruent audio environment. This disrupted their practice and sense of immersion in the virtual environment. Future studies may want to consider using a 360 recorded video of a nature environment and to implement environmental sounds that are congruent with the scene. Furthermore, a possible hypothesis of the negative feedback pertaining to the quality of the virtual environment (e.g., the colors of VR are unable to replicate the naturalness outside) could be due to how some participants were expecting photo-realism in VR. When the world revealed to be animated, this dissonance might have influenced how unreal it felt.

However, not every individual had an immersive experience with virtual reality. Discomfort due to the weight of the headset also impacted participants’ ability to engage in guided practices that are longer than 10 min in duration. Several participants indicated that the headset was too heavy, suggesting that participant’s comfort levels of the headset should be considered. Alternatively, the VR headset may be substituted for other lighter versions of the VR modality such as the Google Cardboard. Such considerations may be important when designing future studies that require lengthy use of the headset.

Limitations of our study include the small sample size, which limits the generalizability of the study to non-clinical and therapeutic settings. Furthermore, our study only involved one session of mindfulness training. Future directions include expanding this study to a large scale randomized controlled trial to evaluate the effectiveness of VR mindfulness with VR hardware that may be more accessible and affordable to the general public. With higher accessibility, future studies should look into longitudinal tracking of home practice and participants’ frequency of use and duration of practice with VR mindfulness.

Other limitations include the suboptimal hardware characteristics of the Oculus Go. Due to limited funding, better and more optimal hardware (e.g., Oculus Quest) was not accessible. Nevertheless, future studies may want to consider the use of optimal hardware as it reduces the risk of developing symptoms of cyber-sickness (Kourtesis et al., 2019). Moreover, software features such as good graphics, spatialized audio, haptics, etc., provide the necessary foundations to induce strong placement and plausibility illusions to significantly reduce the risk of developing VR induced symptoms and effects (Kourtesis et al., 2020). Future studies may want to consider both software and hardware improvements for clinical and research purposes. While participants were informed prior to starting the study that there was a risk of developing symptoms of cyber-sickness and to stop the practice if they experienced it, it was noted that none of the participants reported it. However, it should not be assumed that participants did not experience it. Future studies should include an assessment for cyber-sickness such as the Simulator Sickness Questionnaire (SSQ) or Fast Motion Sickness Score (FMS) (Somrak et al., 2021).

In relation to measuring the sense of presence in VR, some studies suggested that it is more objective to use behavioral (e.g., hand and head movements) and physiological (e.g., heart rate) metrics to measure realism and presence as opposed to just using subjective measures alone (Bailenson et al., 2004; Slater, 2004). However, other studies have suggested that no physiological measure has gathered enough evidence to be reliably used alone without participants evaluating their subjective experience (Grassini and Laumann, 2020). Thus, future studies may want to consider using a combination of both behavioral and physiological metrics in complement to subjective questionnaires of presence.

In conclusion, despite the aforementioned limitations, our study has demonstrated that VR has the potential to be an efficacious supplement to mindfulness training. This study highlights how crucial the type and quality of the virtual environments as well as audio relevance to the environment are to ensure the feeling of presence. Currently, VR mindfulness has not been widely studied within a controlled clinical setting. These results contribute to the emerging literature of interventions aided by VR and are a foundation for future studies to build upon.

## Data Availability Statement

The de-identified data supporting the conclusions of this article will be made available by the authors, upon reasonable request.

## Ethics Statement

The studies involving human participants were reviewed and approved by the SingHealth Centralised Institutional Review Board. The patients/participants provided their written informed consent to participate in this study.

## Author Contributions

DS and CO conceived of the idea and designed the study in collaboration with KD, LJ, SH, and QF. DS and CO collected the data. KD and LJ supervised the study. All authors analyzed the behavioral data, wrote the manuscript, discussed, and commented on the manuscript.

## Conflict of Interest

The authors declare that the research was conducted in the absence of any commercial or financial relationships that could be construed as a potential conflict of interest.

## Footnotes

1
http://www.random.org
