# Corrigendum: Exploring the Use of Virtual Reality for the Delivery and Practice of Stress-Management Exercises

**DOI:** 10.3389/fpsyg.2021.752647

**Published:** 2021-09-08

**Authors:** Desmond Jun Hong Soh, Crystal Huiyi Ong, Qianqian Fan, Denise Ju Ling Seah, Stacey Lee Henderson, Lohsnah Jeevanandam, Kinjal Doshi

**Affiliations:** ^1^Department of Psychology, National University of Singapore, Singapore, Singapore; ^2^Department of Psychology, Singapore General Hospital, Singapore, Singapore

**Keywords:** healthcare professionals, virtual reality, mood, burnout, mindfulness

Lohsnah Jeevanandam was not included as an author in the published article. The corrected Author Contributions Statement appears below.

DS and CO conceived of the idea and designed the study in collaboration with KD, LJ, SH, and QF. DS and CO collected the data. KD and LJ supervised the study. All authors analyzed the behavioral data, wrote the manuscript, discussed, and commented on the manuscript.

The authors apologize for this error and state that this does not change the scientific conclusions of the article in any way. The original article has been updated.

## Publisher's Note

All claims expressed in this article are solely those of the authors and do not necessarily represent those of their affiliated organizations, or those of the publisher, the editors and the reviewers. Any product that may be evaluated in this article, or claim that may be made by its manufacturer, is not guaranteed or endorsed by the publisher.

